# Treatment patterns and survival of patients with locoregional recurrence in early-stage NSCLC: a literature review of real-world evidence

**DOI:** 10.1007/s12032-022-01790-0

**Published:** 2022-10-29

**Authors:** Kathleen Bowes, Nick Jovanoski, Audrey E. Brown, Danilo Di Maio, Rossella Belleli, Shkun Chadda, Seye Abogunrin

**Affiliations:** 1 Genesis Research, Newcastle upon Tyne, UK; 2 F. Hoffmann-La Roche Ltd, Basel, Switzerland

**Keywords:** NSCLC, Locoregional recurrence, Treatments, Overall survival, Progression-free survival

## Abstract

Approximately 10–50% of patients treated for early-stage (I–III), resectable non-small cell lung cancer (eNSCLC) will develop locoregional recurrence. There is a lack of prospective trials evaluating optimal post-surgery follow-up for this patient population, and treatment guidelines recommend salvage therapies such as surgery, local ablative therapy, and (chemo)radiotherapy. A literature review was conducted according to pre-defined criteria to identify observational studies describing treatment patterns and survival outcomes in patients with eNSCLC who experienced locoregional recurrence. Results showed that, in real-world clinical practice, around 80% of patients with locoregional recurrence underwent any form of active treatment. The most frequently administered treatments were chemotherapy (35.7%), chemoradiotherapy (31.2%), radiotherapy (20.3%), and surgery alone (12.8%). Chemoradiotherapy was associated with improved PFS and OS compared with radiotherapy, while no statistically significant survival benefits were observed for patients receiving surgery in addition to these treatments. The overall survival of patients following treatment for locoregional recurrence was generally poor, and the proportion of patients who experienced any form of post-treatment re-recurrence ranged from 35 to 72%. These findings highlight the need to develop more effective treatment strategies for locoregional recurrence, including preventative treatments, and strategies to improve the survival outcomes of those who do develop locoregional recurrence.

**Supplementary Information:**

The online version contains supplementary material available at 10.1007/s12032-022-01790-0.

## Introduction

Lung cancer is the second most common form of cancer, with 2.21 million new cases reported in 2020, and 1.8 million deaths, making it the deadliest form of cancer worldwide [1]. According to histology, lung cancer can be divided into two types: non-small cell, and small cell, where non-small cell lung cancer (NSCLC) makes up around 84% of all lung cancer diagnoses [2].

NSCLC arises mainly in the epithelial cells and can be categorized as adenocarcinoma, squamous cell carcinoma, or large cell cancer [3]. It is staged according to the tumor size, nodes, metastasis (TNM) classification, where all cancers without metastasis are classified as stage I–III, and metastatic cancers are classified as stage IV [4, 5]. Generally, surgically resectable disease is defined as stage I, stage II, and selected stage III tumors where there are no nodes present, or those nodes which are present are benign according to biopsy [6, 7]. Therefore, the preferred method of treatment for NSCLC in the early stages (I–III) typically involves surgical resection with curative intent [8, 9], although there is still a considerable risk of recurrence for these patients [7].

Locoregional recurrence is defined as a recurrence of cancer following curative treatment, at the original tumor site (local), and/or the lymph nodes and tissue in close proximity to the original tumor site (regional) [6]. Rates of locoregional disease recurrence vary greatly between sources, with some studies suggesting a rate of locoregional recurrence of between 10 and 20% for stage I and up to 50% for stage III patients following surgery [10]. Despite this, there is a paucity of research regarding the optimal course of treatment for these patients [7, 11]. Understanding the treatments received by patients with locoregional recurrence, and the survival outcomes for this patient population, is essential to improving the standard of care in this population. The purpose of this review was to identify treatment patterns and modalities in locoregional recurrence, the proportion of patients who receive these treatments, and outcomes related to treatment in real-world clinical practice.

## Methods

### Eligibility criteria

The eligible patient population for this review were patients with early-stage NSCLC who experienced locoregional recurrence. A literature review was conducted to determine the treatment patterns, progression and survival outcomes of these patients in the real-world setting. Searches included a range of relevant interventions and comparators selected according to current treatment practices for eNSCLC, which consists mainly of surgery (often with adjuvant chemotherapy) or SABR in the case of patients considered inoperable [7]. A full list of patient, intervention, comparator, outcomes, and study design (PICOS) criteria used in searches is provided in Table 1.

**Table 1:** PICO elements used to determine studies eligible for inclusion within results of this review

Element	Focus	
Population	Adult patients with early/resectable NSCLC (stage I–III) in the locoregional recurrence health state	
Interventions/comparators	● Atezolizumab ● Pemetrexed ● Nab-paclitaxel ● Gemcitabine ● Vinorelbine ● Nivolumab ● Durvalumab ● Cemiplimab ● Avelumab ● Tegafur ± uracil (UFT) ● Osimertinib	● Pembrolizumab ● Erlotinib ● Cisplatin-based chemotherapy ● Carboplatin-based chemotherapy ● Gefitinib ● Afatinib ● Docetaxel ● Radiotherapy ● Best supportive care ● Etoposide ● Surgery
Outcomes	● Treatment patterns in locoregional recurrence (including palliative and supportive care) ● Survival outcomes for locoregional recurrence	
Study design	Prospective and retrospective observational studies	

*QoL* quality of life

### Outcomes

The majority of studies discussed herein measured progression-free survival (PFS) from the first day of radiotherapy or chemoradiotherapy for locoregional recurrence, to re-recurrence, death, or date of last follow-up [12–15]; one study differed in that PFS was measured from the last day of radiotherapy for locoregional recurrence [16]. Post-recurrence survival (PRS) and overall survival (OS) were both defined as the time from treatment for locoregional recurrence to death or date of last follow-up [12–18].

### Search strategy

Searches were conducted in March and April 2021, with results limited to studies published from 2006 onwards, as studies published before this point were deemed to have less clinical relevance due to the changing treatment landscape. Searches were further restricted to include studies conducted in the following countries: France, Italy, Spain, Germany, the UK, Netherlands, the US, Japan, and South Korea. This review was conducted as part of a broader body of work and these countries were considered relevant to the geographic scope. Initial searches were conducted in Evid AI, using an iterative search strategy. Evid AI is a literature assessment tool, utilizing artificial intelligence (AI) to aid in conducting scientific literature searches [19, 20]. The platform contains over 100 million data points extracted from primary scientific studies and reviews. Sourcing abstracts from PubMed and various conferences, Evid AI uses a patented “machine reading” technology which supports users in finding relevant data. The AI assesses up to 25 million articles per hour, structuring the data to improve the relevance of search results. Broader supplementary searches were conducted in PubMed to identify additional publications relating to treatment patterns, as literature in this area was limited due to the niche nature of the patient population. Database searches were accompanied by searches of conference proceedings from the three most recent meetings of ISPOR, ESMO, ASCO, and WCLC. References cited in included studies were also checked for relevant data.

### Study selection and data extraction

Abstracts were assessed for inclusion by a single reviewer, and the full texts of records identified as relevant were retrieved for further review by a single reviewer, according to the pre-specified PICOS criteria. This work was not a formal systematic literature review, therefore no PRISMA diagram was constructed, and no record of excluded studies and the reasons for exclusion was kept; only included studies were recorded. To assess the survival of patients with locoregional recurrence, the outcomes of interest were progression-free survival and the proportion of patients whose disease progressed or who died as their first event. These data were collected and assessed according to treatment modality. Prioritization criteria were applied to identify the most relevant studies for inclusion (Supplementary Table 1).

Relevant information from included studies was extracted in Microsoft Excel by one reviewer and included details of the study design, baseline characteristics, and the outcomes of interest.

## Results

Searches yielded 1859 publications in total. After screening, 15 publications of real-world evidence studies were prioritised for inclusion describing the treatment options and survival outcomes of early-stage NSCLC patients with locoregional recurrence, all of which were retrospective analyses.

Locoregional recurrence was generally defined as any site of recurrence at the ipsilateral lung and/or regional lymph nodes [21]. Other studies provided more detailed descriptions of the recurrence site, defining the local site as the bronchial stump and adjacent areas, and the regional lymph node as the ipsilateral hilar lymph nodes, mediastinal lymph nodes, and supraclavicular lymph nodes [12, 17]. One study additionally specified that locoregional recurrence should be amenable to local therapy [22]. Patients with local recurrence only were included in one study. However, the definition of recurrence was similar to the above, described as recurrence in the ipsilateral hilar lymph nodes, ipsilateral or contralateral mediastinal lymph nodes, or in the surgical resection margin [14]. Locoregional recurrence was clinically or pathologically confirmed in all included studies.

### Treatment modalities in locoregional recurrence

At a 5-year follow-up of 9001 patients with stage I–III NSCLC who had undergone surgery with curative intent, identified from the US National Cancer database, 1110 patients (12.3%) had developed locoregional recurrence [21]. Of the patients who received active treatment for locoregional recurrence, the most frequently administered treatment was chemotherapy (35.7%), followed by chemoradiotherapy (31.2%), radiotherapy (20.3%), and surgery alone (12.8%) [21]. These data are largely in accordance with data from smaller retrospective single-institution studies conducted in the US, South Korea, and Japan which showed that chemotherapy and chemoradiotherapy were the most commonly administered treatments [17, 21–25].

The type of chemotherapy regimen administered to patients with locoregional recurrence is summarized in Table 2. Platinum-based doublet therapy was reported to be the most commonly used chemotherapy regimen [13–15, 22]

**Table 2:** Chemotherapy regimens administered to patients with locoregional recurrence

Study reference	Chemotherapy regimens reported
Brooks et al. [23]	Platinum/paclitaxel Platinum/pemetrexed Platinum/etoposide Platinum/gemcitabine Pemetrexed alone Paclitaxel alone IL-10 Pembrolizumab Erlotinib
Friedes et al. [22]	Platinum-based sensitising Platinum-based double/ single agent
Terada et al. [14]	Cisplatin/S-1 Cisplatin/vinorelbine Carboplatin/paclitaxel
Lee et al. [15]	Cisplatin/paclitaxel Cisplatin/docetaxel Cisplatin/etoposide Carboplatin/paclitaxel

The type of radiation therapy administered to patients was generally conventional radiotherapy, stereotactic ablative radiotherapy (SABR), brachytherapy [23], or three-dimensional conformal radiotherapy [15]. One study reported that patients with locoregional recurrence received a median biologically equivalent dose (BED) of 79.2 Gy_10_ [15]. These therapies were often administered with concurrent chemotherapy [14–16].

Other, less frequently reported active treatments were thermal ablation, received by 12% of patients at a cancer treatment center in the US [23], and radiofrequency ablation, received by 3% of patients at a hospital in South Korea [24]. An analysis of treatment records at another US cancer center [22] reported that 13.8% and 23.1% of patients received tyrosine kinase inhibitors and immunotherapy, respectively, alongside the main treatment.

Supportive care is a treatment option for patients who are unable or choose not to receive any active treatment. In a study of 128 locoregional recurrence patient records from the Japanese Foundation for Cancer Research, 18% of patients were reported to receive best supportive care [25]. This value approximately agrees with that of a larger retrospective study of US patient records, in which 20.5% of 1110 patients received supportive care [21]. Patients were less likely to receive active treatment if they were older (*p* < 0.001), female (*p* = 0.01), or had either substance abuse (*p* = 0.01), symptomatic recurrence (*p* = 0.045), or ipsilateral lung recurrence (*p* = 0.01) [21].

### Survival of locoregional recurrence patients

#### Progression-free survival

Real-world progression-free survival (PFS) outcomes for patients with locoregional recurrence are summarized in Table 3. Data were taken from retrospective reviews of medical records from single institutions in Japan [12–14], South Korea [15, 16] and Italy [26]. Patients developed locoregional recurrence after surgery with curative intent, with median time to recurrence from primary surgical treatment ranging from 13.6 months [16] to 24 months [26].

**Table 3:** Real-world progression-free survival outcomes by treatment

Study reference	Median follow-up, months	Treatment arm (*n*)	Median, months	2-year, %	5-year, %
Progression-free survival					
Nakamichi et al. [12]	Not reported	Radiotherapy (56)	10.6	25	–
		Chemoradiotherapy (18)	19	44	–
Kim et al. [16]	53.6	Radiotherapy (15)	–	20	–
		Chemoradiotherapy (42)	–	38.2	–
		Radiotherapy, with or without chemotherapy (57)	12.2	33.6	–
Hisakane et al. [13]	30.7	Chemoradiotherapy (40)	20.3	–	–
Lee et al. [15]	25	Chemoradiotherapy (127)	–	34.6	22.3
Agolli et al. [26]	18	Radiotherapy, with or without chemotherapy (28)	20	36.6	–
Terada et al. [14]	48	Radiotherapy, with or without chemotherapy (46)	11	–	22.8
Metastasis-free survival					
Agolli et al. [26]	18	SBRT, with or without chemotherapy (28)	NR	60	–
Kim et al. [16]	53.6	Radiotherapy, with or without chemotherapy (57)	–	47.4	33.5

In most studies, PFS was defined as the time from treatment for locoregional recurrence to re-recurrence or death. Exceptions were Hisakane et al. [13] and Agolli et al. [26], which defined PFS/disease-free survival as the time from treatment to progression or death but did not expressly define progression. There was some variation between studies regarding whether PFS was calculated from the initiation or completion of treatment

*n* number, *SBRT* stereotactic body radiation therapy

Chemoradiotherapy was associated with improved PFS rates compared with radiotherapy in patients who developed locoregional recurrence after curative intent surgery [16, 27]. The 2-year progression-free survival rate and median PFS were 44% and 19 months, respectively, in the chemoradiotherapy arm, and 25% and 10.6 months, respectively, in the radiotherapy arm, according to Nakamichi et al. [12]. In a second study, the 2-year PFS rate was 38.2% in the chemoradiotherapy arm and 20% in the radiotherapy arm [16] (Table 3).

For patients who underwent radiotherapy for locoregional recurrence, PFS outcomes were more favorable for younger patients, patients with one recurrent foci, and patients receiving a higher dose of radiotherapy [15, 16]. On univariate analysis, the 2-year PFS rate was statistically significantly improved for patients aged under 70 years compared with patients aged ≥ 70 years (41.1% vs. 20.0%, *p* = 0.019) and for patients with one recurrent foci compared to those with two or three recurrent foci (45.2% vs. 9.0%, *p* = 0.01) [16]. A second study found that a higher BED_10_ (≥ 79.2 Gy_10_ vs. < 79.2 Gy_10_) was favorable for improved 2-year PFS (40.8% vs. 10.4%, *p* = 0.043) on univariate analysis [15]. No statistically significant differences in the 2-year PFS rate were observed for any other patient characteristics.

After treatment for locoregional recurrence, the proportion of patients who experienced any form of re-recurrence ranged from 35 [18] to 72% [14] (Table 4). Data were taken from retrospective reviews of medical records from single institutions in the US [18], South Korea [24] and Japan [13, 14]; selected patients all had stage I-III disease, with the exception of one study [24] which focused on stage I patients.

**Table 4:** Progression events following treatment for locoregional recurrence

Study reference	Median follow-up, months	Treatment arm (*n*)	Reported outcome	Results No. of patients (%)
Song et al. [24]	37.5	All (36)	Re-recurrence	21 (58)
Terada et al. [14]	48	Radiotherapy, with or without chemotherapy (46)	Recurrence	33 (72)
			Distant recurrence	32 (NA)
Hisakane et al. [13]	30.7	Chemoradiotherapy (40)	Disease relapse	24 (60)
Wu et al. [18]	23	Radiotherapy, with or without chemotherapy (152)	Locoregional failure	64 (42)
			Distant metastasis	53 (35)

#### Overall survival

Median post-recurrence survival was lower for patients who received supportive care compared with those who received active treatment (4.0 vs. 19.9 months, respectively; *p* < 0.001) [21]. These results were from a US-based study of 1,022 patients with locoregional recurrence following complete resection, in which 79.5% of patients received active treatment and the remainder received supportive care [21]. Post-recurrence survival at five years was relatively poor for all patients (4.8% for supportive care only vs. 11.4% for active treatment) [21]. Data from two retrospective reviews of medical records from single institutions based in South Korea [17, 24], showed that for patients receiving any active treatment, 1-year survival rates were 76% and 88%, which dropped to 48% and 55% for 3-year survival.

Chemoradiotherapy was associated with improved overall survival rates compared with radiotherapy in patients who developed locoregional recurrence after curative intent surgery [16, 27]. The 2-year overall survival rate and median OS were 82% and 79.6 months, respectively, in the chemoradiotherapy arm, and 55% and 33.1 months, respectively, in the radiotherapy arm in one study [27]. In a second study, at a median follow-up of 53.6 months, the 2-year overall survival rate and median OS were 73.1% and 86 months, respectively, in the chemoradiotherapy arm, and 33.1% and 20 months, respectively, in the radiotherapy arm [16].

A study of overall survival in South Korean patients who received radiotherapy suggested that a disease-free interval > 1 year was prognostic for improved OS compared with a disease-free interval ≤ 1 year (2-year OS, 81.3% vs. 62.4%, respectively; *p* = 0.009) [24]. However, this association was not found in two other studies [13, 16]

Evidence from another retrospective study of patients in South Korea study suggests that, at three years, there is no statistically significant difference in post-recurrence survival between those who did and did not receive surgery in addition to other therapies for locoregional recurrence (3-year survival, 61% vs. 57%, surgery vs. no surgery, respectively) [24]. At the time of data cut-off, 46% of patients who received surgery had died, as opposed to 39% of patients who did not receive surgery [24]. A full list of survival outcomes by treatment is provided in Table 5.

**Table 5:** Real-world overall survival and deaths following treatment for locoregional recurrence

Study reference	Median follow-up, months	Treatment arm (*n*)	Patient survival rate					
			Median, months	1-year, %	2-year, %	3-year, %	5-year, %	Deaths, *n* (%)
Hisakane et al. [13]	30.7	Chemoradiotherapy (cisplatin and vinorelbine) (40)	65	–	78.9	–	–	18 (45)
Lee et al. [15]	25	Chemoradiotherapy (127)	49	–	72.9	–	43.9	–
Nakamichi et al. [12]	Not reported	Radiotherapy (56)	33.1	–	55	–	35	NA
		Chemoradiotherapy (18)	79.6	–	82	–	53	NA
Kim et al. [16]	53.6	Radiotherapy (15)	20	–	33.3	–	–	–
		Chemoradiotherapy (42)	86	–	73.1	–	–	–
		Radiotherapy, with or without chemotherapy (57)	54.8	–	62.4	–	48.5	–
Wu et al. [18]	23	Radiotherapy, with or without chemotherapy (152)	23	–	49	–	28	–
Agolli et al. [26]	18	Radiotherapy, with or without chemotherapy (28)	31	–	57.5	–	–	9 (32)
Bae et al. [10]	32	Radiotherapy, with or without chemotherapy (64)	18.5	–	47.9	29.5	–	–
Terada et al. [14]	48	Radiotherapy, with or without chemotherapy (46)	50	–	–	–	47.9	–
Wong et al. [21]	60	All active treatment (812)	19.9^a^	–	–	–	11.4^a^	–
		Supportive care (210)	4^a^	–	–	–	4.8^a^	–
Song et al. [24]	37.5	All active treatment (36)	–	88	–	55	–	–
		Surgery, with other treatments (13)	–	–	–	61^a^	–	6 (46)
		No surgery, with other treatments (23)	–	–	–	57^a^	–	9 (39)
Cho et al. [17]		All treatments (38)	–	76^a^	–	48^a^	–	–

^a^Reported as post-recurrence survival

## Discussion

There is a lack of randomized controlled trials in patients with locoregional recurrence, most likely due to the relatively small number of patients who develop only local or regional recurrence [12]. Therefore, the aim of this review was to collate real-world evidence to identify treatment modalities, and subsequent survival outcomes related to these treatments for patients with NSCLC who experience locoregional recurrence.

The largest study identified in this review, comprising 9001 patients from the US National Cancer Database, reported that 12.3% of patients who underwent surgical resection for early-stage NSCLC developed locoregional recurrence [21]. This value is largely in agreement with that reported in other studies [10]. Not all patients who experience locoregional recurrence are suitable for salvage treatment [7]. Real-world data suggested that for patients with locoregional recurrence after initial treatment for early-stage NSCLC, approximately 80% go on to receive any active therapy [21, 25]. Patients of older age, female, or who had substance abuse, symptomatic recurrence, or ipsilateral recurrence were less likely to receive active treatment [21].

For patients who received active therapy, chemotherapy and radiotherapy were the most commonly administered treatments [21]. The proportion of patients who received radiotherapy was comparable to the proportion who were treated with supportive care (20.3 vs. 20.5%, respectively) [21]. Other studies reporting the proportion of patients receiving specific treatment modalities were smaller in size but were largely supportive of these data.

Patients receiving active treatment for locoregional recurrence reported improved survival outcomes compared with patients who received supportive care although 5-year survival rates were generally poor for both treatment arms [21]. Treatment with chemoradiotherapy was associated with improved PFS and OS compared with radiotherapy [12, 16]. Across all included studies, 2-year survival rates for patients treated with chemoradiotherapy ranged from 72.9 [15] to 82% [12]. No differences in survival outcomes were reported when patients received surgery in combination with chemotherapy or radiotherapy compared with patients who received chemotherapy or radiotherapy alone [24].

The included studies investigated patients with locoregional recurrence following surgical resection, with the exception of Brooks et al. [23]. Prior to locoregional recurrence, patients in this study were treated with stereotactic ablative radiotherapy (SABR), as they were not suitable candidates for surgery. Patients who are ineligible for surgery are likely to have poorer survival outcomes; outcomes from this study should therefore be compared to other studies with caution.

The survival data identified in this review are limited by a number of factors. All included studies were retrospective in nature and, with the exception of the study by Wong et al. [21] populations of the studies were generally small; although the smallest study had a population of 28 patients [26], when patients were grouped by treatment, the smallest sub-population consisted of only 13 patients [24]. Many of the studies were conducted in Japan and South Korea, and while survival data were largely consistent between studies, these results may not be representative of patients in other geographic regions. Studies reporting survival data covered a limited number of therapies; of the studies presented in this review that investigated radiotherapy as a treatment option, only two reported separate outcomes for patients who did and did not receive concurrent chemotherapy [12, 16]. Only one study reported survival outcomes following surgery for locoregional recurrence [24]. Other studies did not stratify outcomes by treatment, but rather gave the overall post-recurrence survival of patients who received any type of treatment for locoregional recurrence. Many studies only had a single treatment arm.

Limited data were found on the types and dosage of radiation therapy, and the chemotherapy regimens administered to patients with locoregional recurrence. It is not clear whether any of the patients who underwent active treatment also received palliative treatment, or what types of palliative treatment were available to them. Supportive care was the only type of palliative treatment for which data were obtained.

Overall, real-world survival data shows that the prognosis of patients with locoregional recurrence is generally poor, highlighting the need for more research into preventative treatments to limit the number of patients who develop locoregional recurrence, as well as more efficacious treatment strategies to improve outcomes of those who do develop locoregional recurrence.

## Conclusion

Treatment guidelines for patients with early-stage NSCLC and locoregional recurrence recommend salvage therapy for this patient population [7]. Real-world evidence suggests that the majority of patients who receive any active treatment for locoregional recurrence are treated with chemotherapy or chemoradiotherapy [21], and survival outcomes tend to be more favorable for patients who undergo chemoradiotherapy [12, 16]. No statistically significant survival benefits were observed for patients receiving surgery in addition to these treatments, although evidence regarding surgery was limited [24]. Best supportive care was administered to approximately 20% of patients with locoregional recurrence [21, 25]. However, it was not clear whether these patients were ineligible for active treatment or if they chose not to receive treatment. Further studies are warranted to assess optimal treatment strategies for patients with eNSCLC, including adjuvant treatments for the prevention of locoregional recurrence, and strategies to improve survival of patients who go on to develop locoregional recurrence.

## Supplementary Information

Below is the link to the electronic supplementary material.

Supplementary file1 (DOCX 19 KB)
