# Supplementary material for: Treatment patterns and survival of patients with locoregional recurrence in early-stage NSCLC: a literature review of real-world evidence
Source: Med Oncol. 2022 Oct 29;40(1):4. doi: 10.1007/s12032-022-01790-0 (PMC9617826; doi:10.1007/s12032-022-01790-0)
Supplement: Supplementary file 1 — Supplementary file1 (DOCX 19 KB) [file 12032_2022_1790_MOESM1_ESM.docx]

Supplementary Table 1: Prioritization criteria for inclusion of studies

| **To be prioritized** | **To be de-prioritized** |
| --- | --- |
| Articles that are primarily about the topics of interest listed in the inclusion criteria | Articles that are partly or tangentially about these topics |
| Primary studies that have larger study populations | Primary studies that have smaller study populations |
| Primary studies that have longitudinal designs and longer follow-up times | Primary studies that have cross-sectional designs or shorter follow-up times |
| Articles published more recently | Older articles |
| Articles published in higher-tier journals, e.g., with higher impact factors | Articles published in lower-tier journals, e.g., with lower impact factors |
